# Bile Acid Metabolome after an Oral Lipid Tolerance Test by Liquid Chromatography-Tandem Mass Spectrometry (LC-MS/MS)

**DOI:** 10.1371/journal.pone.0148869

**Published:** 2016-02-10

**Authors:** Andreas Schmid, Hannah Neumann, Thomas Karrasch, Gerhard Liebisch, Andreas Schäffler

**Affiliations:** 1 Department of Internal Medicine III, Giessen University Hospital, Giessen, Germany; 2 Institute of Clinical Chemistry and Laboratory Medicine, Regensburg University Hospital, Regensburg, Germany; Beckman Research Institute of City of Hope, UNITED STATES

## Abstract

**Context:**

Besides their role in intestinal resorption of lipids, bile acids are regarded as endocrine and metabolic signaling molecules. The detailed profile of bile acid species in peripheral blood after an oral lipid tolerance test (OLTT) is unknown.

**Objective:**

We quantified the regulation of 18 bile acids after OLTT in healthy individuals.

**Material and methods:**

100 volunteers were characterized by anthropometric and laboratory parameters and underwent OLTT. Venous blood was drawn in the fasted state (0 h) and at 2h, 4h, and 6 h after OLTT. Serum concentrations of 18 bile acids were measured by LC-MS/MS.

**Results:**

All of the 6 taurine-conjugated bile acids (TUDCA, THDCA, TCA, TCDCA, TDCA, TLCA) and all of the 6 glycine-conjugated bile acids (GUDCA, GHDCA, GCA, GCDCA, GDCA, GLCA) rose significantly at 2h and remained elevated during OLTT. Of the primary bile acids, CA remained unchanged, whereas CDCA significantly decreased at 4h. Of the secondary bile acids, DCA, UDCA and HDCA were not altered, whereas LCA decreased. There was a significant positive correlation between the intestinal feed-back regulator of bile acid synthesis FGF-19 and bile acids. This correlation seems to depend on all of the six taurine-conjugated bile acids and on GCA, GDCA, and GCDCA. Females and users of hormonal contraception displayed higher levels of taurine-conjugated bile acids.

**Conclusions:**

The novelty of the study is based on the identification of single bile acids during OLTT. LC-MS/MS-based quantification of bile acids in serum provides a reliable tool for future investigation of endocrine and metabolic effects of bile acids.

## Introduction

Beyond their established role in facilitating the intestinal resorption of lipids and beyond the well-known mechanism of ileal reabsorption and enterohepatic recirculation to the liver, there is increasing data on pleiotropic and systemic effects of bile acids. Thus, bile acids have been shown to affect carbohydrate metabolism, lipid metabolism, immune regulation, growth and differentiation [1–3]. Based on this observation, it is intriguing to speculate that bile acids circulating in the peripheral blood might act as endocrine and metabolic signaling molecules upon food intake. Regarding metabolic function, postprandial regulation of systemic bile acids might be of importance but has been only poorly investigated.

Bile acids exert their effects via two main pathways, the small heterodimeric partner (SHP)-dependent nuclear receptor farnesoid X receptorα (FXRα) pathway and the SHP-independent G-protein-coupled bile acid receptor (TGR5) pathway. Thus, bile acid effects resemble those of hormones. Bile acids act as ligands to FXRα. In the liver, FXRα-activated target gene transcription controls bile acid synthesis, insulin-sensitivity, glycolysis, gluconeogenesis, lipogenesis, carbohydrate metabolism and lipid metabolism via SHP- and sterol regulatory element binding protein-1c (SREBP-1c)-dependent mechanisms [4–7].

Fibroblast growth factors (FGFs) represent a family of 22 proteins resembling the function of classical hormones acting on FGF receptors (FGFR1-4). Whereas a dominant role of FXRα-regulated FGF-19 in the control of bile acid metabolism has been widely accepted, a physiological role of peroxisome proliferator-activated receptor-α (PPARα)-regulated FGF-21 in metabolic homeostasis is less clear [8–10]. Tissue-specific metabolic activities of FGF-19 and FGF-21 are modulated by beta Klotho, a homologous single-pass transmembrane protein binding to FGF receptors, and by particular FGF receptor isoform expression [11].

FGF-19 has been regarded as a regulator of hepatic bile acid synthesis [12, 13]. FGF-19 gene transcription and secretion by enterocytes of the ileum is induced by FXRα activation upon bile acid binding. FGF-19 then circulates to the liver via the portal vein and downregulates hepatic bile acid synthesis by a feedback inhibitory circuit [7, 14] through fibroblast growth factor receptor-4 (FGFR-4)-mediated transcriptional repression of the enzyme cholesterol 7 alpha-hydroxylase (CYP7A1), the rate-limiting enzyme in bile acid synthesis [15]. Bile acids as FXRα activators are able to induce hepatic expression and secretion of FGF-21 [16].

Key FXR pathways have been described in multiple animal models and include molecular and pathogenetic implications for hepatic fibrosis, inflammation, atherosclerosis, lipid metabolism, carbohydrate metabolism, endothelial function, thyroid function and bile acid homeostasis [17].

In a metabolic and endocrine point of view, alterations of peripheral blood bile acid concentrations can be investigated by mixed meals given to individuals. However, mixed meals containing proteins, carbohydrates and lipids in different combinations represent a disadvantage when interpreting data. Recently, our group has investigated the isolated effect of carbohydrates on bile acid concentrations by an oral glucose tolerance test [18]. However, there is no single study available investigating the isolated effect of orally ingested lipids on systemic bile acid concentrations in healthy individuals.

Therefore, it was the aim of the present study to investigate by LC-MS/MS

- whether 18 primary, secondary and tertiary conjugated and non-conjugated bile acids are altered in serum of n = 100 healthy individuals during an oral lipid tolerance test (OLTT) by using a carbohydrate- and protein-free lipid solution
- whether anthropometric parameters influence the time course of bile acids after OLTT
- whether alterations of the serum bile acid metabolome during OLTT correlates to variations in serum FGF-19 levels

## Material and Methods

### Study cohort

The present study cohort has been characterized earlier for anthropometric and laboratory parameters as well as for the regulation of adipokines and FGF-19/FGF-21 upon OLTT [19, 20]. Briefly, participants were examined at the University Hospital of Regensburg. 100 healthy volunteers (58 females and 42 males; 66 with normal weight and 34 overweight/obese) gave their written informed consent to the study approved by the ethical committee of the Medical Faculty, University of Regensburg. Exclusion criteria were a positive history of any kind of illness, evidence of acute or chronic infection within 10 days prior to the OLTT, age < 18 years or > 55 years, and any kind of medication (except oral contraceptives). Pregnant and menstruating women were not admitted to the study. Age, BMI, hip circumference, waist circumference, waist/hip ratio, triceps skinfold thickness, and blood pressure were recorded. Patients’ history regarding type 2 diabetes and cardiovascular diseases as well as smoking habits or hormonal contraception were documented. Table 1 summarizes the predominant characteristics of the study population as published earlier in detail [19].

**Table 1:** Demographic and clinical statistics of the study population.

Study population (n = 100)	
Age (years)	28.1 ± 7.7 [18–54]
Males n *(%)*	42 (42)
Females n *(%)*	58 (58)
*Anthropometric parameters*	
Mean BMI (kg/m^2^)	24.2 ± 5.0 [14.8–46.1]
BMI < 25 kg/m^2^ (n, %)	66 (66)
BMI ≥ 25 kg/m^2^ (n, %)	34 (34)
Waist circumference (cm)	83.9 ± 13.0 [63.0–122.0]
Hip circumference (cm)	99.5 ± 10.1 [77.0–131.0]
Waist-Hip Ratio	0.84 ± 0.09 [0.68–1.07]
Skin fold thickness (mm)	11.3 ± 8.9 [1.0–47.0]
*Circulation*	
Systolic blood pressure (mm Hg)	121 ± 15 [90–174]
Diastolic blood pressure (mm Hg)	78 ± 9 [60–105]
Heart rate (min ^-1^)	73 ± 12 [50–105]
*Carbohydrate-Metabolism*	
Plasma glucose 0h (mg/dl)	74 ± 13 [40–110]
Plasma insulin 0h (mU/l)	8.9 ± 5.7 [2.3–33.3]
Plasma C-peptide 0h (μg/l)	1.3 ± 0.5 [0.5–3.8]
*Lipoprotein-Metabolism*	
Total cholesterol 0h (mg/dl)	191 ± 39 [52–297]
Triglycerides 0h (mg/dl)	120 + 50 [24–299]
LDL cholesterol 0h (mg/dl)	111 ± 33 [21–196]
HDL cholesterol 0h (mg/dl)	60 ± 17 [23–107]
Inflammation	
CRP (mg/l)	3.9 ± 3.2 [2.9–27]

Data are presented as means ± SD (standard deviation) and ranges (square brackets). Demographic data of the present study cohort were under investigation earlier and were reported elsewhere in detail [19, 20]. BMI was not correlated significantly with bile acid or FGF-19 levels. There was a marginal, physiologically irrelevant correlation between age and free bile acid concentration (p = 0.049; r = -0.200).

### Oral lipid tolerance test (OLTT)

OLTT was conducted after an overnight fast of 12 h. During OLTT, the participants had to rest and not to eat or smoke. The ingestion of 500 ml of pure water was allowed over the 6 hours. Venous blood samples were drawn at 0h (fasting), 2h, 4h, and 6 h. Serum was prepared by 10 min of centrifugation at 4000 rotations per min (rpm). As reported recently by our group in detail [19], we used a solution that was free of proteins and carbohydrates. Briefly, the OLTT solution (160 ml; 758.1 kcal; 75 g vegetable fat as triglycerides, 9.2 g fatty acids as pure vegetable oils) comprised the following components: *Component 1*: 150 ml water/fat solution (Calogen^R^ NUTRICIA-Neutral, Pfrimmer Nutricia, Erlangen, Germany): 75 g vegetable fat as pure triglycerides containing 7.95 g saturated fatty acids, 45.6 g mono-unsaturated fatty acids, 21.45 g polyunsaturated fatty acids, 69 g water, 0.15 g carbohydrates (insignificant trace amount), 675 kcal. *Component 2*: 5 ml (4.6 g; 41.5 kcal) sun flower oil containing 10.2% saturated fatty acids, 26.3% mono-unsaturated fatty acids, and 63.5% poly-unsaturated fatty acids. *Component 3*: 5 ml (4.6g; 41.6 kcal) olive oil containing 14% saturated fatty acids, 78% mono-unsaturated fatty acids, and 8% poly-unsaturated fatty acids.

### Quantification of human bile acids in serum by HPLC-MS/MS

Bile acid species were quantified as described previously by LC-MS/MS [21]. Briefly, serum samples were spiked with a mixture of deuterated bile acids as internal standard prior to protein precipitation. Serum extracts were subjected to LC-MS/MS detection in negative ion mode after base-line separation of isobaric species. The LC-MS/MS system consisted of an API 4000 QTrap (AB Sciex, Darmstadt, Germany) that was coupled with electrospray ionization. Chromatographic separation was achieved by an Agilent 1200 HPLC system (Agilent, Waldbronn, Germany). Quantification was achieved using a matrix calibrator generated by standard addition.

### Statistical analysis

For calculating mean values ± standard deviation (± SD) or ± standard error of the mean (± SEM), a statistical software package (SPSS 22.0) was used. Mean values were compared by the non-parametric Mann-Whitney U-test for 2 independent samples and the Kruskal-Wallis-H-test for *k* independent samples. For subgroup analysis of mean values, Bonferroni´s correction was performed. Correlation analysis was done by using the Spearman-Rho test for linear variables. A p-value below 0.05 (two tailed) was considered as statistically significant. In the figures, the circles are showing the mean values and the whiskers are giving the 95% confidence interval (CI) of the mean.

## Results

### Effect of OLTT on main bile acid species

Table 2 gives an overview of the main bile acid species and their systemic response in serum during OLTT. All of the main bile acid species, total/free bile acids, primary/secondary bile acids, and taurine-/glycine-conjugated bile acids show significant alterations during OLTT. Total bile acids increased (~3fold) with a maximum at 2h and remained elevated at 4h and 6h when compared to the fasted state. In contrast, free bile acids decreased significantly (~50%) at 6h after OLTT. Both, primary and secondary bile acid species significantly increased (~3-fold) with a maximum at 2h and remained at higher levels when compared to the fasted state. Similarly, the taurine- and glycine-conjugated bile acids significantly increased (~4 to 5-fold) with a maximum level at 2h and also remained at a higher plateau when compared to 0h.

**Table 2:** Main human bile acid species in serum before and during oral lipid tolerance test (OLTT) measured by HPLC-MS/MS.

Bile acid species (nmol/l)	OLTT	OLTT	OLTT	OLTT	p-Value
	0h	2h	4h	6h	Kruskal-Wallis-H test
Total bile acids	3308.720 ± 392.252	9947.405 ± 823.842**	7919.28 ± 795.655**	5516.245 ± 471.777**	p<0.001
Free bile acids	1321.010 ± 235.997	1219.923 ± 171.397	745.489 ± 81.044	648.024 ± 77.715*	p<0.001
Primary bile acids	1989.724 ± 285.873	6266.021 ± 544.104**	4767.497 ± 471.335**	3281.506 ± 295.014**	p<0.001
Secondary bile acids	1318.995 ± 125.796	3681.389 ± 334.222**	3151.780 ± 348.645**	2234.746 ± 200.073**	p<0.001
Taurine-conjugated bile acids	360.374 ± 41.359	1796.152 ± 227.605**	1573.599 ± 284.211**	1023.587 ± 169.335**	p<0.001
Glycine-conjugated bile acids	1627.333 ± 191.093	6931.332 ± 608.544**	5600.190 ± 564.251**	3844.633 ± 341.230**	p<0.001

Data are given as mean concentrations ± SEM (standard error of the mean) in nmol/l. For overall correlation analysis of data, the non-parametric Kruskal-Wallis-H test was applied. For direct comparison of mean bile acid levels from 0h to 2h, 4h and 6h respectively, the non-parametric Mann-Whitney U-Test was used (* p = 0.001; ** p<0.001). Since 4 subgroups (points of time) were compared, significance is reached at a p-value < 0.0125 due to Bonferroni´s correction.

### Quantitative profiling of 18 human single bile acids during OLTT

Since changes in main bile acid species do not allow a deeper insight into bile acid physiology and potential endocrine effects of circulating single/specific bile acids after oral lipid ingestion, 18 bile acids were quantified at all points of time (0h, 2h, 4h, 6h) during OLTT in all participating individuals (n = 100). Table 3 summarizes the individual changes of each of the 18 bile acids during OLTT.

**Table 3:** Profile of 18 human bile acid species in serum before and during oral lipid tolerance test (OLTT) measured by HPLC-MS/MS.

Bile acid species (nmol/l)	OLTT 0h	OLTT 2h	OLTT 4h	OLTT 6h	p-Value Kruskal-Wallis test
Primary bile acids					
Non-conjugated					
CA	873.479 ± 204.050	898.947 ± 424.138	465.679 ± 148.421	481.794 ± 177.428	0.2542
CDCA	569.232 ± 132.654	453.927 ± 72.115	258.656 ± 45.992*	261.614 ± 50.172	0.001
Taurine-conjugated					
TCA	69.491 ± 11.138	353.543 ± 58.511**	291.204 ± 66.445**	192.564 ± 41.567**	<0.001
TCDCA	188.461 ± 20.865	922.491 ± 104.140**	787.482 ± 114.169**	531.767 ± 74.298**	<0.001
Glycine-conjugated					
GCA	321.045 ± 52.853	1370.278 ± 140.969**	995.840 ± 111.553**	712.917 ± 85.597**	<0.001
GCDCA	741.949 ± 92.486	3099.537 ± 273.912**	2474.421 ± 238.492**	1672.319 ± 138.267**	<0.001
Secondary bile acids					
Non-conjugated					
DCA	403.422 ± 36.961	534.504 ± 54.941	400.323 ± 40.126	341.823 ± 30.930	0.065
HDCA	51.293 ± 11.781	50.838 ± 4.923	42.022 ± 4.159	36.430 ± 3.067	0.088
LCA	142.273 ± 53.182	128.878 ± 18.523*	103.149 ± 17.075	82.148 ± 11.409	0.004
Taurine-conjugated					
TDCA	80.859 ± 11.849	428.328 ± 71.909**	412.929 ± 103.339**	241.241 ± 54.809**	<0.001
THDCA	5.040 ± 0.893	22.608 ± 4.483**	21.426 ± 4.720**	13.582 ± 2.259**	<0.001
TLCA	7.595 ± 1.339	34.069 ± 6.728**	32.182 ± 7.081**	20.204 ± 3.360**	<0.001
Glycine-conjugated					
GDCA	353.597 ± 46.483	1438.036 ± 145.168**	1193.556 ± 143.518**	787.910 ± 85.000**	<0.001
GHDCA	126.382 ± 17.910	378.963 ± 61.405**	356.581 ± 63.921**	268.137 ± 38.275**	<0.001
GLCA	162.795 ± 25.813	521.973 ± 87.772**	489.985 ± 91.349**	363.635 ± 54.707**	<0.001
Tertiary bile acids					
Non-conjugated					
UDCA	142.273 ± 53.182	128.878 ± 18.523	103.149 ± 17.075	82.148 ± 11.409	0.195
Taurine-conjugated					
TUDCA	7.595 ± 1.339	34.069 ± 6.728**	32.182 ± 7.081**	20.204 ± 3.360**	<0.001
Glycine-conjugated					
GUDCA	162.795 ± 25.813	521.973 ± 87.772**	489.985 ± 91.349**	363.635 ± 54.707**	<0.001

Data are given as mean concentrations ± SEM (standard error of the mean) in nmol/l. For overall correlation analysis of data, the non-parametric Kruskal-Wallis-H test was applied. For direct comparison of mean bile acid levels from 0h to 2h, 4h, and 6h respectively, the non-parametric Mann-Whitney U-Test was used (* p<0.01; ** p<0.001). Since 4 subgroups (timepoints) were compared, significance is reached at a p-value < 0.0125 due to Bonferroni´s correction. Primary bile acids: CA, cholic acid; CDCA, chenodeoxycholic acid. Secondary bile acids: DCA, deoxycholic acid, HDCA, hyodeoxycholic acid. LCA, lithocholic acid; Tertiary bile acids: UDCA, ursodeoxycholic acid; Taurine-conjugated bile acids: TCA, taurocholic acid; TCDCA, taurochenodeoxycholic acid; TDCA, taurodeoxycholic acid; TLCA, taurolithocholic acid; TUDCA, tauroursodeoxycholic acid; THDCA, taurohyodeoxycholic acid. Glycine-conjugated bile acids: GCA, glycocholic acid; GCDCA, glycochenodeoxycholic acid; GDCA, glycodeoxycholic acid; GLCA, glycolithocholic acid; GUDCA, glycoursodeoxycholic acid; GHDCA, glycohyodeoxycholic acid.

*Taurine-conjugated bile acids*: All of the 6 taurine-conjugated bile acids (TUDCA, THDCA, TCA, TCDCA, TDCA, TLCA) rose significantly (~5-fold) and early (at 2h) and remained at higher levels until the end of the OLTT.

*Glycine-conjugated bile acids*: All of the 6 glycine-conjugated bile acids (GUDCA, GHDCA, GCA, GCDCA, GDCA, GLCA) rose significantly (~3 to 4-fold) and early (at 2h) and remained at higher levels until the end of the OLTT.

*Primary bile acids*: Of the two primary bile acids (CA and CDCA), CA remained unchanged during OLTT, whereas CDCA significantly decreased at 4h.

*Secondary bile acids*: Of the secondary bile acids, DCA and HDCA were not significantly altered during OLTT. LCA decreased during OLTT in a stepwise manner. However, significance was only reached at 2h when compared to 0h.

*Tertiary bile acids*: Of the tertiary bile acids, UDCA was not altered significantly.

**Fig 1:**
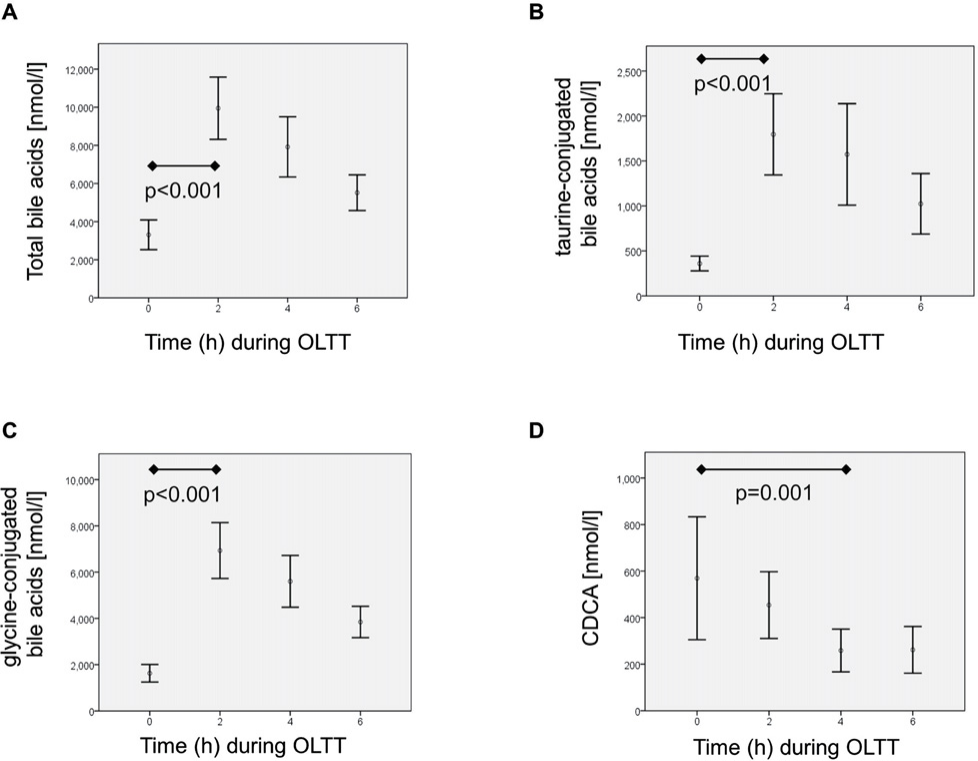
Time-dependent variations of bile acids during an oral lipid tolerance test (OLTT) are shown in healthy individuals (n = 100) for total bile acids (A), taurine-conjugated bile acids (B), glycine-conjugated bile acids (C), and chenodeoxycholic acid (D). The bars are showing the median values and the whiskers are giving the 95% confidence interval (CI) of the mean. CDCA, chenodeoxycholic acid.

Fig 1 exemplarily depicts the time course of total bile acids, taurine- and glycine-conjugated bile acids as well as CDCA.

### Correlation analysis of postprandial FGF-19 and FGF-21 serum concentrations with bile acid species after oral lipid ingestion

Table 4 summarizes the results of the correlation analysis of postprandial FGF-19 concentrations with bile acid species. Our previous work [20] demonstrated a significant (p = 0.006) increase of triglycerides between 2h and 4h (Table 1) and a significant rise of FGF-19 at 4h during OLTT in this cohort [20]. In detail, basal FGF-19 levels were 105 ± 81 pg/ml and rose up to 141 ± 102 pg/ml (p = 0.006) at 4 h [20]. In comparison, the maximum rise of bile acids occurred early at 2h during OLTT and the 1–2h delay between the postprandial rise of bile acids and FGF-19 is in accordance with data from the literature [18, 22]. However, this time kinetics has not yet been proved for a pure lipid tolerance test devoid of carbohydrates and proteins.

**Table 4:** Correlation analysis of postprandial FGF-19 serum concentrations with bile acid species.

		p-value	correlation coefficient r
	Bile acid species		
FGF-19	total bile acids	<0.001	+ 0.41
FGF-19	free bile acids	0.012	+ 0.25
FGF-19	primary bile acids	0.001	+ 0.34
FGF-19	secondary bile acids	<0.001	+ 0.7
FGF-19	taurine-conjugated bile acids	<0.001	+ 0.8
FGF-19	glycine-conjugated bile acids	0.001	+ 0.34
	Single bile acids		
FGF-19	TUDCA	0.012	+ 0.25
FGF-19	THDCA	0.012	+ 0.25
FGF-19	TCA	0.024	+ 0.23
FGF-19	GCA	0.026	+ 0.23
FGF-19	TCDCA	0.002	+ 0.30
FGF-19	GCDCA	0.002	+ 0.31
FGF-19	TDCA	<0.001	+ 0.4
FGF-19	GDCA	<0.001	+ 0.37
FGF-19	DCA	0.003	+ 0.3
FGF-19	TLCA	0.004	+ 0.29

Levels of FGF-19 (pg/ml) drawn at 6h after oral lipid ingestion were correlated with bile acid species (nmol/ml) at 6h by the Spearman-Rho test. No significant correlations were found for GUDCA, UDCA, GHDCA, HDCA, CA, CDCA, GLCA, and LCA (data not shown).

In the present study, we could detect significant positive correlations between FGF-19 levels and bile acids which were most striking at 6h after lipid ingestion. With respect to main bile acid species, there was a highly significant and positive correlation between FGF-19 levels and total bile acids, free bile acids, primary bile acids, secondary bile acids, taurine-conjugated bile acids and glycine-conjugated bile acids. Since it was our aim to identify those bile acids that are mainly responsible for these class effects, correlation analysis was performed for each of the 18 bile acids measured. No significant correlations were found for GUDCA, UDCA, GHDCA, HDCA, CA, CDCA, GLCA, and LCA. The positive correlation between FGF-19 and bile acids was strongest for the taurine-conjugated bile acids (r = +0.8). All of the six taurine-conjugated bile acids TUDCA, THDCA, TCA, TCDCA, TDCA, and TLCA showed this positive correlation with FGF-19. The correlation between FGF-19 and the glycine-conjugated bile acids is caused only by GCA, GDCA, and GCDCA, since GUDCA, GHDCA, and GLCA had no effect. For interested readers, the correlation analysis of FGF-19 with bile acid species is presented for gender subgroups and for contraceptive users/non-users under *Online Supporting Information* (S1 and S2 Tables). However, subgroups might be too small for profound or extensive interpretation.

In our previous study [20], FGF-21 levels were shown to decrease significantly and transiently during OLTT. In detail [20], basal FGF-21 concentrations decreased from 158.8 ± 203.6 pg/ml at 0h down to 127.6 + 184.4 pg/ml at 2h (p = 0.03). We now retrieved these data and calculated a correlation analysis for FGF-21 and bile acids. In contrast to FGF-19 mentioned above, FGF-21 was negatively correlated to some of the bile acids. In detail, FGF-21 concentrations at 6h were significantly and negatively correlated with taurine-conjugated bile acids in total (p = 0.017; r = -0.24) and with TCA (p = 0.002; r = -0.3), TDCA (p = 0.014; r = -0.25), and TLCA (p = 0.04; r = -0.21).

### Effects of gender and BMI on postprandial bile acid concentration

Subgroup analysis displayed differential effects of sex on postprandial bile acid levels at 6h after OLTT. Table 5 summarizes the effects of gender on postprandial bile acids. Taurine-conjugated bile acids in general were significantly higher in females when compared to males (~2.5-fold). In detail, all of the taurine-conjugated bile acid species were significantly higher (~1.5 to 3-fold) in females when compared to males. Since bile acids were not significantly different between males and females at baseline (data not shown), these sex differences are not due to differences in overall levels but possibly to differences in sensitivity to lipid challenge. None of the glycine-conjugated bile acids was gender-dependent. UDCA was the only bile acid identified with significantly lower levels in females when compared to males. The remaining bile acids were not significantly different between females and males. When individuals were subdivided into lean subjects (BMI <25 kg/m^2^) and overweight/obese subjects (BMI ≥ 25 kg/m^2^), mean values of all bile acids measured were not significantly different (data not shown). Importantly, when taurine-conjugated bile acid levels of males were compared to those of women not using contraceptives, we could not detect significant changes between genders. Thus, the effect of hormonal contraception was further investigated.

**Table 5:** Effects of gender and of hormonal contraception on bile acid concentrations.

Bile acids (nmol/l)	females	males	Significance
	n = 58	n = 42	p-value
Taurine-conjugated bile acids (all)	1374.403 ± 278.042	539.126 ± 81.016	0.001
TUDCA	23.505 ± 4.527	15.645 ± 4.968	0.023
THDCA	15.923 ± 3.059	10.405 ± 3.312	0.016
TCA	270.666 ± 69.171	84.71 ± 16.072	0.001
TCDCA	680.584 ± 119.578	326.257 ± 50.081	0.002
TDCA	351.110 ± 91.460	89.517 ± 15.582	<0.001
TLCA	33.470 ± 5.592	13.562 ± 1.518	0.002
Bile acid	Contraception (females)	Contraception(females)	Significance
	Yes (n = 38)	No (n = 20)	p-value
Taurine-conjugated bile acids (all)	1780.774 ± 405.625	602.300 ± 126.166	0.001
TUDCA	25.711 ± 3.999	19.315 ± 10.848	0.003
THDCA	17.147 ± 2.667	13.474 ± 7.592	0.005
TCA	356.897 ± 102.082	106.825 ± 30.228	0.003
TCDCA	860.032 ± 172.146	33.635 ± 73.599	0.002
TDCA	480.629 ± 134.704	105.025 ± 27.824	<0.001
TLCA	40.363 ± 7.903	19.684 ± 4.402	0.046

Bile acids (nmol/l) with significantly different concentrations between females/males and users/non-users of hormonal contraception at 6h during OLTT are summarized. Mean values ± SEM are shown.

### Effects of hormonal contraception on postprandial bile acid concentration

Users of hormonal contraception displayed differential effects regarding their postprandial bile acid levels at 6h after OLTT. Table 5 summarizes the effects of hormonal contraception on postprandial bile acids. Taurine-conjugated bile acids were higher in users when compared to non-users (~3-fold). In detail, all of the taurine-conjugated bile acid species were affected by contraceptives and showed significantly higher concentrations. Since taurine-conjugated bile acids were also slightly higher (p = 0.026; data not shown) at baseline in users vs. non-users, the marked differences at 6h after lipid ingestion might be due to differences in overall levels as well as to differences in sensitivity to lipid challenge. CDCA was the only bile acid identified whose concentrations were significantly lower under hormonal contraception. The remaining bile acids were not significantly different between users and non-users of hormonal contraception (data not shown).

## Discussion

The present study was conducted in order to gain systematic insight into changes of serum bile acids and their regulator proteins FGF-19 and FGF-21 during OLTT in healthy individuals. Since the uptake of bile acids by the liver via the enterohepatic circulation is highly efficient, nano/micro-molar fractions enter the systemic circulation and might exert yet unknown endocrine and metabolic effects on organs other than the liver. Systemic bile acid concentrations might result from a passive spillover by the enterohepatic circulation (portal vein) or from direct hepatic release via the hepatic veins. The latter is a matter of speculation but would offer the fascinating hypothesis of bile acids as hepatokines exerting multi-organ systemic effects. For example, molecular studies have shown that some bile acid species are able to interact with lipid bilayers at sub-micellar concentrations [23]. Thus, disturbance of liposomal membranes by bile acids might affect a plethora of ligand-receptor interactions and subsequent signal transduction as well as intracellular protein trafficking.

The novelty of the present study is based on the identification of single bile acids that are responsible for the systemic alterations of main bile acid species such as total/free bile acids, primary/secondary bile acids, and taurine-/glycine-conjugated bile acids during OLTT. This was only possible by applying a sophisticated technical approach for the measurement of 18 different human bile acids. HPLC-MS/MS is suitable for rapid and precise quantification of human bile acids, as shown earlier by our group [18]. Whereas all of the main bile acid species showed alterations during OLTT, it is now possible to detect single bile acids that cause these species effects and to exclude single bile acids not contributing to the postprandial systemic alterations. Moreover, the present study including n = 100 participants is large enough to be statistically robust against outliers when compared to similar studies with less participants [24–26].

SHP-independent bile acid signaling can be investigated by targeting FGF-19. Our group and others have shown that serum levels of FGF-19 rise significantly 4h after oral lipid ingestion [20] and this response is blunted in patients with non-alcoholic fatty liver disease and insulin resistance [24]. We observed a rapid onset increase of bile acids after 2h during OLTT, similar to a bile acid increase at 1h after a glucose challenge [18]. After this increase, bile acids remained significantly elevated up to 6h when compared to the fasted state. The postprandial increase of bile acids is followed by a rise of FGF-19 after 90–120 min [22] in normal subjects. Since we could recently describe a significant rise of FGF-19 at 4h after OLTT in the presented cohort of healthy volunteers [20], we retrieved these data and tested for a possible correlation of basal and postprandial FGF-19 levels with each of the bile acids measured. Whereas basal FGF-19 and bile acid concentrations showed no correlation in the fasted state, we were able to describe significant positive correlations of postprandial (6h) FGF-19 levels with a distinct pattern of bile acids. A widely accepted mechanism for the postprandial FGF-19 rise in plasma is based on the release of FGF-19 from enterocytes following bile acid binding to FXRα and its activation. Since FGF-19 plasma levels rise significantly later (after 4h upon OLTT) when compared to plasma bile acids (after 2h) [20, 22], the positive correlation between FGF-19 and postprandial bile acids might indicate the existence of an alternative route of FGF-19 secretion upon oral lipid uptake. It currently remains unclear why only a distinct type of bile acids is correlated postprandially with FGF-19 whereas others are not. Future studies have to address this interesting question. Since fatty liver disease and insulin resistance are known factors [24] that are able to blunt FGF-19 secretion, an individual metabolic constellation might affect the correlation between bile acids and FGF-19 release.

In contrast, FGF-21 concentrations significantly declined during OLTT and there was a negative correlation between FGF-21 and taurine-conjugated bile acids during OLTT. These results obtained *in vivo* challenge the results obtained in primary hepatocytes *in vitro* [16], where a bile acid-induced and FXRα-dependent secretion of FGF-21 has been reported. The exact mechanism and the physiological background how and why bile acids change after OLTT remain unclear. There are two principle mechanisms for a postprandial increase of systemic bile acids, a spillover phenomenon from the intestinal circulation to the systemic circulation or a direct release by the liver. Since FGF-19 is upregulated typically late after 4h during OLTT, bile acids are upregulated relatively fast after 2h. This might argue against a spillover phenomenon from the intestinal circulation and favors a direct release by the liver upon oral lipid uptake. The latter potential mechanism might provide the potential basis for a role of bile acids as “hepatokines” that has to be investigated by further mechanistic studies. The observed decrease of free bile acid concentrations contrary to conjugated bile acid species suggests an elevated turnover rate of free bile acid conjugation with glycine or taurine, respectively, as a consequence of oral lipid ingestion. As discussed earlier by Matysik et al. investigating bile acid profiles during an oral glucose tolerance test [18], postprandial FXRα activation by bile acids might induce a rise in bile acid amidation rate. However, since neither the rate of bile acid resorption from the small intestine nor of hepatic bile acid synthesis was quantified in the presented study, no proof of principle can be given for this speculation.

Not all the bile acid species measured activate the receptors FXR and TGR5 with the same potency. This fact complicates the interpretation of data implying that different bile acids patterns might have different biological impact according to receptor activation and signaling.

Taken together, after a pure lipid ingestion the time kinetics of bile acids, triglycerides and FGF-19 in healthy individuals could be clarified by the present study. Bile acids are upregulated very fast after 2h of ingestion. This rise in bile acids precedes the increase of triglycerides (Table 1) which typically occurs between 2h and 4h. After 4h, FGF-19 becomes upregulated. Whereas there is an established and unquestioned role of the enterohepatic circulation as a route for bile acids and FGF-19, future studies have to investigate the exact routes of bile acids, FGF-19, and FGF-21 from the intestine and/or form the liver into the systemic circulation. It would be of interest to conduct studies on a cellular level investigating whether postprandially upregulated bile acids and FGF-19 differentially regulate the activation of beta Klotho in a given metabolic context such as hyperlipidemia or hyperglycemia.

### Summary

- Total bile acids as well as taurine- and glycine-conjugated bile acids show an early (2h) rise after OLTT and remain elevated up to 6h. This bile acid rise in peripheral blood precedes the postprandial rise of FGF-19 seen at 4h and the postprandial rise of triglycerides seen between 2h to 4h.
- Of the primary bile acids, CA remained unchanged after OLTT, whereas CDCA decreased at 4h.
- Of the secondary bile acids, LCA decreased and the others remained unchanged.
- There is a significant and positive correlation between FGF-19 and several bile acids. The strongest correlation was documented for taurine-conjugated bile acids.
- There is a significant and negative correlation between FGF-21 and taurine-conjugated bile acids.
- Higher levels of taurine-conjugated bile acids in females are caused by hormonal contraception whereas glycine-conjugated bile acids were not affected by gender or contraception

## Conclusions

To our knowledge, this is the first study quantifying 18 different bile acid species in peripheral blood of healthy individuals by LC-MS/MS after oral lipid ingestion by using a solution free of carbohydrates and proteins. Moreover, the novelty of the present study lies on the identification of single bile acids that are responsible for the systemic alterations of main bile acid species. LC-MS/MS-based quantification of bile acids in peripheral blood serum provides a reliable tool for future investigation of endocrine and metabolic effects of circulating bile acids beyond their function in enterohepatic circulation and liver physiology.

## Supporting Information

S1 Table Correlation analysis of postprandial FGF-19 serum concentrations with bile acid species in gender subgroups. (DOC)

S2 Table Correlation analysis of postprandial FGF-19 serum concentrations with bile acid species in subgroups of hormonal contraception. (DOC)

## References

[1] Fiorucci S, Cipriani S, Baldelli F, Mencarelli A. Bile acid-activated receptors in the treatment of dyslipidemia and related disorders. Progress in lipid research. 2010;49(2):171–85. 10.1016/j.plipres.2009.11.001 . 19932133

[2] Fiorucci S, Cipriani S, Mencarelli A, Renga B, Distrutti E, Baldelli F. Counter-regulatory role of bile acid activated receptors in immunity and inflammation. Current molecular medicine. 2010;10(6):579–95. . 20642438 10.2174/1566524011009060579

[3] Fiorucci S, Mencarelli A, Distrutti E, Palladino G, Cipriani S. Targetting farnesoid-X-receptor: from medicinal chemistry to disease treatment. Curr Med Chem. 2010;17(2):139–59. . 19941473 10.2174/092986710790112666

[4] Prawitt J, Staels B. Bile acid sequestrants: glucose-lowering mechanisms. Metabolic syndrome and related disorders. 2010;8 Suppl 1:S3–8. 10.1089/met.2010.0096 . 20977365

[5] Stroeve JH, Brufau G, Stellaard F, Gonzalez FJ, Staels B, Kuipers F. Intestinal FXR-mediated FGF15 production contributes to diurnal control of hepatic bile acid synthesis in mice. Laboratory investigation; a journal of technical methods and pathology. 2010;90(10):1457–67. 10.1038/labinvest.2010.107 . 20531290 PMC6643294

[6] Staels B, Handelsman Y, Fonseca V. Bile acid sequestrants for lipid and glucose control. Current diabetes reports. 2010;10(1):70–7. 10.1007/s11892-009-0087-5 20425070 PMC2821506

[7] Holt JA, Luo G, Billin AN, Bisi J, McNeill YY, Kozarsky KF, et al Definition of a novel growth factor-dependent signal cascade for the suppression of bile acid biosynthesis. Genes & development. 2003;17(13):1581–91. 10.1101/gad.1083503 12815072 PMC196131

[8] Angelin B, Larsson TE, Rudling M. Circulating fibroblast growth factors as metabolic regulators—a critical appraisal. Cell metabolism. 2012;16(6):693–705. 10.1016/j.cmet.2012.11.001 . 23217254

[9] Kharitonenkov A. FGFs and metabolism. Current opinion in pharmacology. 2009;9(6):805–10. 10.1016/j.coph.2009.07.001 . 19683963

[10] Cicione C, Degirolamo C, Moschetta A. Emerging role of fibroblast growth factors 15/19 and 21 as metabolic integrators in the liver. Hepatology. 2012;56(6):2404–11. 10.1002/hep.25929 . 22753116

[11] Kurosu H, Choi M, Ogawa Y, Dickson AS, Goetz R, Eliseenkova AV, et al Tissue-specific expression of betaKlotho and fibroblast growth factor (FGF) receptor isoforms determines metabolic activity of FGF19 and FGF21. The Journal of biological chemistry. 2007;282(37):26687–95. 10.1074/jbc.M704165200 17623664 PMC2496965

[12] Beenken A, Mohammadi M. The FGF family: biology, pathophysiology and therapy. Nature reviews Drug discovery. 2009;8(3):235–53. 10.1038/nrd2792 19247306 PMC3684054

[13] Jones S. Mini-review: endocrine actions of fibroblast growth factor 19. Molecular pharmaceutics. 2008;5(1):42–8. 10.1021/mp700105z . 18179175

[14] Inagaki T, Choi M, Moschetta A, Peng L, Cummins CL, McDonald JG, et al Fibroblast growth factor 15 functions as an enterohepatic signal to regulate bile acid homeostasis. Cell metabolism. 2005;2(4):217–25. 10.1016/j.cmet.2005.09.001 . 16213224

[15] Goodwin B, Jones SA, Price RR, Watson MA, McKee DD, Moore LB, et al A regulatory cascade of the nuclear receptors FXR, SHP-1, and LRH-1 represses bile acid biosynthesis. Molecular cell. 2000;6(3):517–26. . 11030332 10.1016/s1097-2765(00)00051-4

[16] Cyphert HA, Ge X, Kohan AB, Salati LM, Zhang Y, Hillgartner FB. Activation of the farnesoid X receptor induces hepatic expression and secretion of fibroblast growth factor 21. The Journal of biological chemistry. 2012;287(30):25123–38. 10.1074/jbc.M112.375907 22661717 PMC3408207

[17] Adorini L, Pruzanski M, Shapiro D. Farnesoid X receptor targeting to treat nonalcoholic steatohepatitis. Drug discovery today. 2012;17(17–18):988–97. 10.1016/j.drudis.2012.05.012 . 22652341

[18] Matysik S, Martin J, Bala M, Scherer M, Schaffler A, Schmitz G. Bile acid signaling after an oral glucose tolerance test. Chemistry and physics of lipids. 2011;164(6):525–9. 10.1016/j.chemphyslip.2011.05.003 . 21679700

[19] Karrasch T, Leszczak S, Bala M, Ober I, Martin J, Schmid A, et al Short-term regulation of Visfatin release in vivo by oral lipid ingestion and in vitro by fatty acid stimulation. Exp Clin Endocrinol Diabetes. 2014;122(2):126–34. 10.1055/s-0033-1363262 . 24554513

[20] Schmid A, Leszczak S, Ober I, Karrasch T, Schaffler A. Short-term and divergent regulation of FGF-19 and FGF-21 during oral lipid tolerance test but not oral glucose tolerance test. Exp Clin Endocrinol Diabetes. 2015;123(2):88–94. 10.1055/s-0034-1395635 . 25654672

[21] Scherer M, Gnewuch C, Schmitz G, Liebisch G. Rapid quantification of bile acids and their conjugates in serum by liquid chromatography-tandem mass spectrometry. Journal of chromatography B, Analytical technologies in the biomedical and life sciences. 2009;877(30):3920–5. 10.1016/j.jchromb.2009.09.038 . 19819765

[22] Lundasen T, Galman C, Angelin B, Rudling M. Circulating intestinal fibroblast growth factor 19 has a pronounced diurnal variation and modulates hepatic bile acid synthesis in man. Journal of internal medicine. 2006;260(6):530–6. 10.1111/j.1365-2796.2006.01731.x . 17116003

[23] Yang L, Feng F, Fawcett JP, Tucker IG. Kinetic and equilibrium studies of bile salt-liposome interactions. Journal of liposome research. 2015;25(1):58–66. 10.3109/08982104.2014.928888 . 24960448

[24] Schreuder TC, Marsman HA, Lenicek M, van Werven JR, Nederveen AJ, Jansen PL, et al The hepatic response to FGF19 is impaired in patients with nonalcoholic fatty liver disease and insulin resistance. American journal of physiology Gastrointestinal and liver physiology. 2010;298(3):G440–5. 10.1152/ajpgi.00322.2009 . 20093562

[25] Zhao X, Peter A, Fritsche J, Elcnerova M, Fritsche A, Haring HU, et al Changes of the plasma metabolome during an oral glucose tolerance test: is there more than glucose to look at? American journal of physiology Endocrinology and metabolism. 2009;296(2):E384–93. 10.1152/ajpendo.90748.2008 . 19066319

[26] Shaham O, Wei R, Wang TJ, Ricciardi C, Lewis GD, Vasan RS, et al Metabolic profiling of the human response to a glucose challenge reveals distinct axes of insulin sensitivity. Molecular systems biology. 2008;4:214 10.1038/msb.2008.50 18682704 PMC2538910

