# Supplementary material for: Bile Acid Metabolome after an Oral Lipid Tolerance Test by Liquid Chromatography-Tandem Mass Spectrometry (LC-MS/MS)
Source: PLoS One. 2016 Feb 10;11(2):e0148869. doi: 10.1371/journal.pone.0148869 (PMC4749208; doi:10.1371/journal.pone.0148869)
Supplement: S1 Table — (DOC) [file pone.0148869.s001.doc]

|  |  | **Females (n=58)** | | **Males (n=42)** | |
| --- | --- | --- | --- | --- | --- |
|  |  | **p** | **r** | **p** | **r** |
|  | **Bile acid species** |  |  |  |  |
| FGF-19 | total bile acids | 0.004 | 0.37 | 0.003 | 0.44 |
| FGF-19 | free bile acids | 0.220 |  | 0.026 | 0.34 |
| FGF-19 | primary bile acids | 0.014 | 0.32 | 0.019 | 0.36 |
| FGF-19 | secondary bile acids | 0.009 | 0.34 | 0.009 | 0.40 |
| FGF-19 | taurine-conjugated bile acids | 0.016 | 0.32 | 0.128 |  |
| FGF-19 | glycine-conjugated bile acids | 0.009 | 0.34 | 0.018 | 0.37 |
|  | Single bile acids |  |  |  |  |
| FGF-19 | TUDCA | 0.177 |  | 0.051 |  |
| FGF-19 | GUDCA | 0.346 |  | 0.037 | 0.32 |
| FGF-19 | UDCA | 0.603 |  | 0.032 | 0.46 |
| FGF-19 | THDCA | 0.189 |  | 0.048 | 0.31 |
| FGF-19 | GHDCA | 0.381 |  | 0.043 | 0.31 |
| FGF-19 | HDCA | 0.553 |  | 0.106 |  |
| FGF-19 | TCA | 0.078 |  | 0.748 |  |
| FGF-19 | GCA | 0.087 |  | 0.373 |  |
| FGF-19 | CA | 0.556 |  | 0.544 |  |
| FGF-19 | TCDCA | 0.068 |  | 0.202 |  |
| FGF-19 | GCDCA | 0.025 | 0.30 | 0.010 | 0.39 |
| FGF-19 | CDCA | 0.246 |  | 0.087 |  |
| FGF-19 | TDCA | 0.001 | 0.42 | 0.097 |  |
| FGF-19 | GDCA | 0.001 | 0.43 | 0.086 |  |
| FGF-19 | DCA | 0.026 | 0.29 | 0.069 |  |
| FGF-19 | TLCA | 0.053 |  | 0.261 |  |
| FGF-19 | GLCA | 0.040 | 0.27 | 0.235 |  |
| FGF-19 | LCA | 0.48 |  | 0.570 |  |

**S1 Table: Correlation analysis of postprandial FGF-19 serum concentrations with bile acid species in gender subgroups.** Levels of FGF-19 drawn at 6h after oral lipid ingestion were correlated with bile acid species at 6h by the Spearman-Rho test; r = correlation coefficient
