# Supplementary material for: Bile Acid Metabolome after an Oral Lipid Tolerance Test by Liquid Chromatography-Tandem Mass Spectrometry (LC-MS/MS)
Source: PLoS One. 2016 Feb 10;11(2):e0148869. doi: 10.1371/journal.pone.0148869 (PMC4749208; doi:10.1371/journal.pone.0148869)
Supplement: S2 Table — (DOC) [file pone.0148869.s002.doc]

|  |  | **Contraception (females)** | | **Contraception**  **(females)** | |
| --- | --- | --- | --- | --- | --- |
|  |  | **Yes (n=38)** | | **No (n=20)** | |
|  |  | **p** | **r** | **p** | **r** |
|  | **Bile acid species** |  |  |  |  |
| FGF-19 | total bile acids | 0.015 | 0.39 | 0.289 |  |
| FGF-19 | free bile acids | 0.526 |  | 0.121 |  |
| FGF-19 | primary bile acids | 0.040 | 0.34 | 0.251 |  |
| FGF-19 | secondary bile acids | 0.015 | 0.39 | 0.613 |  |
| FGF-19 | taurine-conjugated bile acids | 0.045 | 0.33 | 0.356 |  |
| FGF-19 | glycine-conjugated bile acids | 0.011 | 0.41 | 0.424 |  |
|  | Single bile acids |  |  |  |  |
| FGF-19 | TUDCA | 0.104 |  | 0.895 |  |
| FGF-19 | GUDCA | 0.336 |  | 0.885 |  |
| FGF-19 | UDCA | 0.144 |  | 0.494 |  |
| FGF-19 | THDCA | 0.092 |  | 0.748 |  |
| FGF-19 | GHDCA | 0.366 |  | 0.980 |  |
| FGF-19 | HDCA | 0.905 |  | 0.466 |  |
| FGF-19 | TCA | 0.096 |  | 0.663 |  |
| FGF-19 | GCA | 0.060 |  | 0.940 |  |
| FGF-19 | CA | 0.667 |  | 0.482 |  |
| FGF-19 | TCDCA | 0.103 |  | 0.387 |  |
| FGF-19 | GCDCA | 0.039 | 0.34 | 0.442 |  |
| FGF-19 | CDCA | 0.051 |  | 0.457 |  |
| FGF-19 | TDCA | 0.007 | 0.43 | 0.214 |  |
| FGF-19 | GDCA | 0.002 | 0.48 | 0.274 |  |
| FGF-19 | DCA | 0.299 |  | 0.040 | 0.48 |
| FGF-19 | TLCA | 0.110 |  | 0.266 |  |
| FGF-19 | GLCA | 0.111 |  | 0.205 |  |
| FGF-19 | LCA | 0.600 |  | 0.667 |  |

**S2 Table: Correlation analysis of postprandial FGF-19 serum concentrations with bile acid species in subgroups of hormonal contraception.** Levels of FGF‑19 drawn at 6h after oral lipid ingestion were correlated with bile acid species at 6h by the Spearman-Rho test; r = correlation coefficient
